# New Insights Into Pathophysiology of β-Thalassemia

**DOI:** 10.3389/fmed.2022.880752

**Published:** 2022-04-12

**Authors:** Maria Sanchez-Villalobos, Miguel Blanquer, Jose M. Moraleda, Eduardo J. Salido, Ana B. Perez-Oliva

**Affiliations:** ^1^Hematology Service, Virgen de la Arrixaca University Hospital, Murcia, Spain; ^2^Biomedical Research Institute of Murcia (IMIB), Murcia, Spain; ^3^Centro de Investigaci3n Biomédica en Red de Enfermedades Raras (CIBERER), Madrid, Spain

**Keywords:** anemia, thalassemia, β-globin, GATA1, inflammasome

## Abstract

β-thalassemia is a disease caused by genetic mutations including a nucleotide change, small insertions or deletions in the β-globin gene, or in rare cases, gross deletions into the β-globin gene. These mutations affect globin-chain subunits within the hemoglobin tetramer what induces an imbalance in the α/β-globin chain ratio, with an excess of free α-globin chains that triggers the most important pathogenic events of the disease: ineffective erythropoiesis, chronic anemia/chronic hypoxia, compensatory hemopoietic expansion and iron overload. Based on advances in our knowledge of the pathophysiology of β-thalassemia, in recent years, emerging therapies and clinical trials are being conducted and are classified into three major categories based on the different approach features of the underlying pathophysiology: correction of the α/β-globin disregulation; improving iron overload and reverse ineffective erythropoiesis. However, pathways such as the dysregulation of transcriptional factors, activation of the inflammasome, or approach to mechanisms of bone mineral loss, remain unexplored for future therapeutic targets. In this review, we update the main pathophysiological pathways involved in β-thalassemia, focusing on the development of new therapies directed at new therapeutic targets.

## Introduction

Thalassemias is an inherited hemoglobin disorder characterized by reduced or absent globin chain synthesis, resulting in variable clinical phenotypes from severe chronic anemia requiring lifelong transfusion and iron chelating therapy to asymptomatic individuals (1).

Traditionally, β-thalassemias have been more common in countries in the Mediterranean area, North and Central Africa, Southeast Asia, and the Middle East. However, as a result of migrations of populations, β-thalassemias are now encountered in other regions, such as Northern Europe and North America (2).

**Figure 1:**
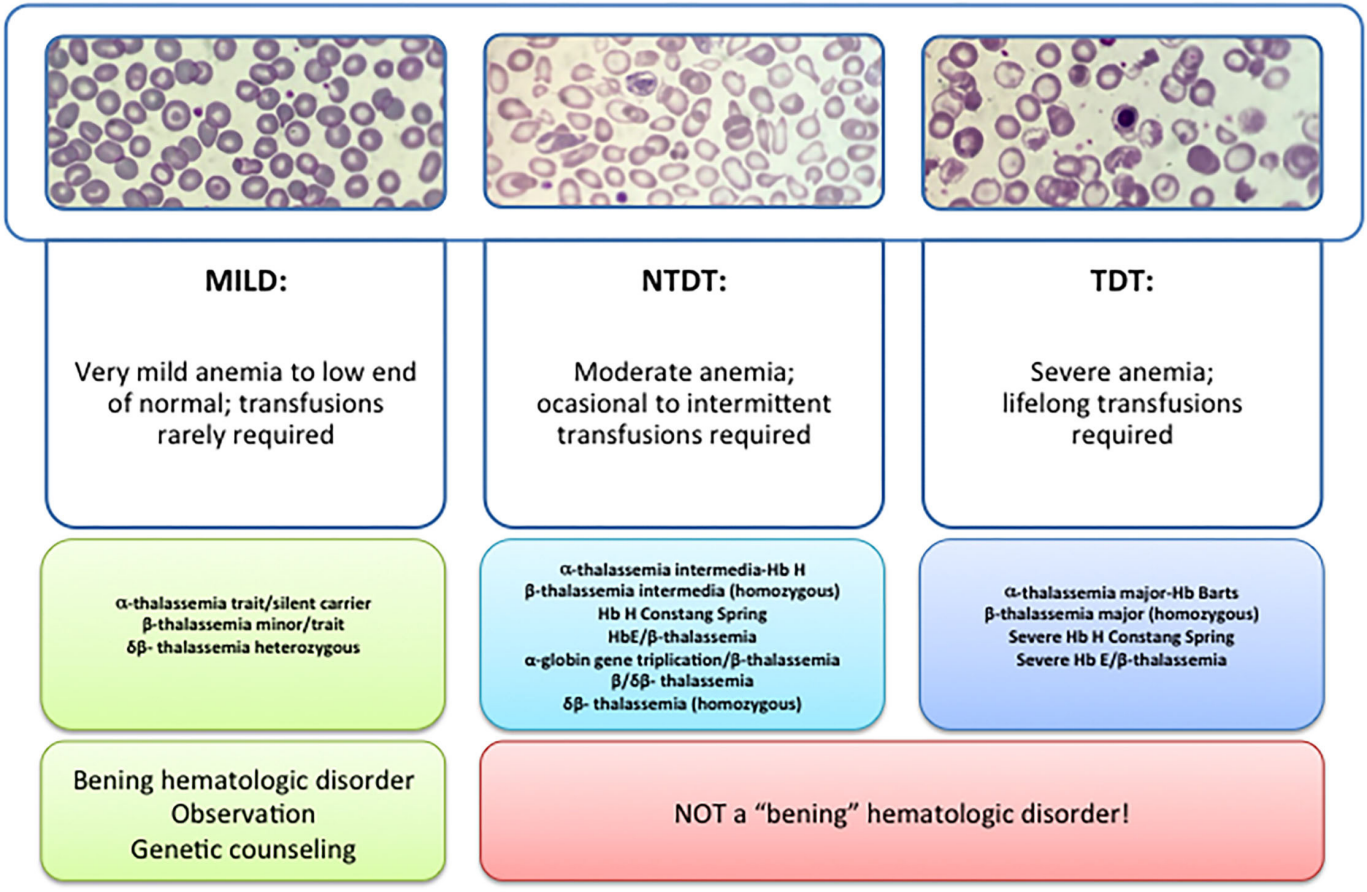
Types of thalassemia. Genotype–Phenotype Association. α and β-thalassemias are genetically heterogeneous diseases. The clinical management with RBC transfusions is an essential factor in classifying them as either transfusion-dependent thalassemia (TDT) or non–transfusion-dependent thalassemia (NTDT). Patients with TDT need life-long regular transfusions for survival in early childhood while patients with NTDT do not need life-long regular transfusions for survival and normally have later in childhood or even in adulthood with mild/moderate anemia that requires only occasional or short-course regular transfusions under concrete clinical circumstances during times of erythroid stress (infection, pregnancy, surgery, or aplastic crisis); however, usually present the typical complications of TDT such as extramedullary hematopoiesis, iron overload, leg ulcers, and osteoporosis. Patients with TDT include those with β-thalassemia major or severe forms of β-thalassemia intermedia, HbE/β-thalassemia, or α-thalassemia/HbH disease. NTDT mainly encompasses three clinically distinct forms: β-thalassemia intermedia (β-TI), hemoglobin E/β- thalassemia (mild and moderate forms), and α-thalassemia intermedia (hemoglobin H disease).

β -thalassemia has a broad clinical spectrum, and traditionally has been classified in the clinic in thalassemia major (TM), thalassemia intermedia (TI), and thalassemia minor (Figure 1). The TM grouped patients with more severe anemia from an early age who require periodic blood transfusions associated with iron chelation for life, while thalassemia minor, the less severe manifestation, is characterized by people with mild asymptomatic anemia and a heterozygous condition (trait) for thalassemia. TI constituted a group with a variable clinical spectrum, from mild to moderate to moderately severe anemia, who do not require blood transfusions on a regular basis, sometimes only occasionally, but who do develop various complications of thalassemia such as extramedullary hematopoiesis, pulmonary hypertension, iron overload, leg ulcers, skeletal deformities, and growth retardation (3, 4).

Recently, this classification has changed due to better understanding of the pathophysiology of the disease and findings focused on the clinical management and complications of IT that show that these patients may present with the same serious complications as transfused patients later in life. In 2012, the International Thalassemia Federation adopted the new terminology for clinical classification of transfusion-dependent thalassemia (TDT) and non–transfusion-dependent thalassemia (NTDT) that groups in three different types in the clinic: α-thalassemia intermedia (hemoglobin H disease) and β-thalassemia intermedia (β-TI), hemoglobin E/β- thalassemia (mild and moderate forms). Differentiating a new patient with thalassemia as TDT or NTDT is essential and requires an accurate clinician's evaluation using various indicators such as hematological parameters, particularly baseline Hb levels, and follow-up for a minimum of 3 to 6 months to determine clinical severity is recommended before making a diagnosis of TDT or NTDT (5, 6).

The three important pathophysiologic factors in β-thalassemias are: chronic anemia/hypoxia, ineffective erythropoiesis, and iron overload. The harshness of the disease depends mainly on molecular deficiencies. Chain imbalance causes excess unstable α chains to provoke within erythroid progenitors, leading to cell membrane decline and cell lysis. This triggers an alteration in the mycomedial environment of the bone marrow due to an imbalance of cytokines that causes the erythroid progenitors to proliferate but with inadequate maturation, which is called ineffective erythropoiesis. This cytokine imbalance together with bone marrow hyperplasia causes extramedullary erythropoiesis and subsequently the associate bone deformations. Because of anemia/chronic hypoxia, infective erythropoiesis retroelements, is maintained and perpetuates over time (3, 6).

## Ineffective Erythropoiesis

**Figure 2:**
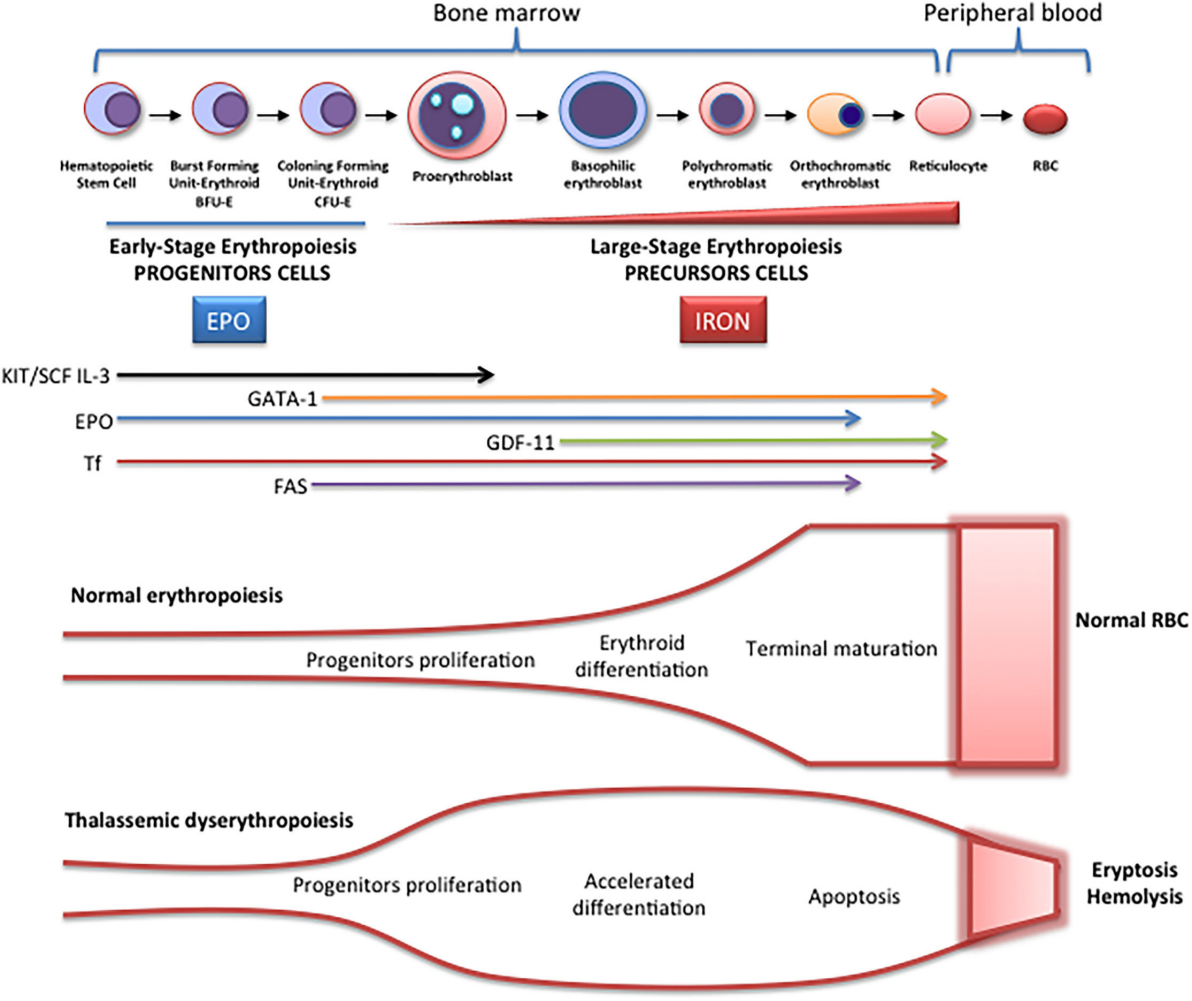
Schematic representation of erythropoiesis. During erythroid development, several stages occur in which a complex network of molecules are expressed (EPO, iron, transcription factors) are involved. In early-stage erythropoiesis, EPO is the main regulator after BFU-E formation. In this stage, GATA-1 promotes erythropoiesis and increases the EPO receptor expression. In large-stage erythropoiesis molecules such as transferrin and growth/differentiating factor 11 (GDF11) are involved. Erythroid expansion is negatively regulated by association of FAS to FAS ligand, which has as a consequence the apoptosis on immature erythroid cells, and GDF11 and other members of the TGF-β family, which negatively regulate erythrocyte differentiation and maturation from the early to the late stages.

Erythropoiesis is a finely regulated process in which every stage is highly regulated by different signal transduction pathways and proteins. Erythropoiesis process in humans is divided into two parts: the early stage of erythropoiesis and the late stage. The first stage is EPO-dependent whereas the second stage is iron-dependent. Erythropoietin (EPO-dependent stage) is the main regulator of early-stage erythropoiesis whereas erythrocyte differentiation and maturation are negatively regulated by the transforming growth factor (TGF-family), the late-stage erythropoiesis or iron-dependent stage of erythropoiesis (Figure 2).

### EPO-Dependent Erythropoiesis

In this stage, EPO is fundamental for the proliferation of erythroid progenitors. The recognition of EPO by EPO-R on the surface of these precursors induces JAK2 activation activating STAT5 phosphorylation, with associated induction of erythroid antiapoptotic genes and increase of erythroid progenitors proliferation and survival (7). The production of EPO and the expansion of erythropoiesis is regulated according to demand (hypoxia, hemorrhage, hemolysis). On the contrary, if the production of erythrocytes is adequate, down-regulation by a mechanism of apoptosis is performed. The predisposition of EPO-dependent erythroid progenitors to apoptosis is associated with different levels of FAS protein (CD95) expression and the FAS ligand (FASL), which belong to the family of TNF receptors, the binding of which activates the caspase cascade and consequently, the immature cell apoptosis limiting erythropoiesis expansion. On the other hand, if the need for erythrocytes increases, EPO production increases, and apoptosis is reduced because EPO stimulates the production of heat shock protein 70 (HSP70) that protects GATA1 from cleavage by FAS/FASL and the activation of the caspase cascade (8).

### Fe-Dependent Erythropoiesis

In the large stage, the presence of iron is essential for the synthesis of hemoglobin, and the integrity of the erythroferrone (ERFE)-hepcidin-ferroportin axis is essential for iron homeostasis (9, 10). ERFE is a potent negative regulator of hepcidin. When it is chronically elevated, such as in situations of ineffective erythropoiesis, low plasma iron availability occurs. Transferrin (and its cellular receptor) is also involved in this stage as well as growth differentiation factors like GDF11, a member of the TGF-β superfamily, which negatively regulate erythrocyte maturation and differentiation (11).

TGF-β receptor ligands are a group of cytokines that include TGF, activins, bone morphogenetic proteins (BMPs), and GDF-11 and play an important role in the regulation of erythropoiesis within the hematopoietic stem cell niche. Various activins, and in particular GDF-11, exert inhibitory activity at the late stage of erythropoiesis.

In the TGF signaling pathway, ligand binding to the type II receptor leads to the recruitment and phosphorylation of the type I receptor and phosphorylation of regulatory SMADs (R-SMADs), SMAD2 and 3, to form the R-SMAD/SMAD4 complex, which modulates the expression of target genes inducing an inhibitory activity on erythroid differentiation by inducing apoptosis in erythroblasts.

During normal erythroid maturation, reduced GDF11 expression with consequent TGF- β signaling suppression, and EPO stimulation occur in parallel, and both are essential for the differentiation of hematopoietic erythroid progenitor cells (12).

In β-thalassemia, ineffective erythropoiesis is triggered by two main pathogenic mechanisms (7). On the one hand, α-globin chains aggregates sequester cytosolic heat shock protein 70 (HSP70). This inhibits its nuclear translocation and protects the erythroid transcription factor GATA-factor 1 (GATA1) from cleavage. On the other hand, these toxic aggregates of α-globin chains stimulate the formation of radical oxygen species (ROS) (whose formation is also produced by other mechanisms such as iron overload), which activate GDF11, which in turn activates the inhibitory pathway of SMAD2/3, and as a consequence, erythroid differentiation is inhibited (13).

## Iron Overload

Iron overload is one of the main pathogenic events in β-thalassemia. Apart from the transfusion-dependent iron overload in patients with TDT, there is a mechanism by which an inappropriate increase in intestinal iron absorption occurs, both in patients with TDT and in NTDT.

This mechanism is triggered by ineffective erythropoiesis. The accumulation of erythroid precursors during ineffective erythropoiesis increases the production of erythropherrone, potent negative regulator of hepcidin secreted by bone marrow erythroblasts (10), which negatively regulates the expression of hepcidin. The decrease in hepcidin, the main negative ferroportin modulator (an iron transporter protein in the basolateral membrane of the enterocyte), increases iron absorption (hepcidin inhibits iron absorption and recycling ferroportin) and release of iron from the reticuloendothelial in situations of iron overload and iron sequestration in erythropoiesis. It has been described that iron availability during stress erythropoiesis is produced by increase of ERFE (13).

In NTDT, growth differentiation factor 15 (GDF-15) can induce hepcidin downregulation. This is a member of the transforming growth factor- (TGF-β) family which use to be upregulated during ineffective erythropoiesis, causing the down regulation of hepcidin (14).

## Bone Disease in β-Thalassemia

Recently, several advances in the understanding of the pathophysiology of bone disease in β-thalassemia have been done. Classically, it was directly attributed to ineffective erythropoiesis and secondary bone expansion, but recently it has been shown that there is an imbalance of cytokines that can directly alter bone metabolism, although the mechanisms involved are not yet well-established.

Both patients with TDT and NTDT show marked decreases in bone mineral density (BMD), despite optimization of transfusions, and low BMD continues to be a frequent complication in these patients.

The mechanisms that have been postulated to explain the loss of bone mineral density in patients with β-thalassemia include explicit effects of abnormal erythroid proliferation with bone expansion, increased circulating erythropoietin (EPO), iron bone deposit with iron toxicity, and oxidative stress with endocrine secondary disorders (hypogonadism, deficit GH-IGF-1, vitamin D deficiency) that which in turn affect the bone mineral loss and secondary osteoporosis (15).

In recent years, there is growing evidence of the relationship between erythropoiesis, bone mineral metabolism and iron homeostasis. Recently, a mechanism responsible for the activation of osteoclasts in thalassemic patients has been described that could be associated to cytokine dysregulation and, in particular, to the modification of the RANK/RANKL/OPG axis, which is essential for the regulation of osteoclastogenesis (16, 17). The OPG/RANK/RANKL system is essential for the regulation of osteoclastogenesis. Osteoprotegerin (OPG) or osteoclastogenesis inhibition factor (OCIF or TNFRSF11B), is a member of the superfamily of tumor necrosis factor (TNFR) receptors that is expressed and secreted in numerous tissues (lung, heart, kidneys, liver, intestine, stomach, brain, thyroid gland and spinal cord) as well as in bone in which its main function is to inhibit the maturation and activation of osteoclasts.

Recently, it has been shown ERFE binds and sequesters some members of the bone morphogenetic protein (BMP) family, primarily BMP2, BMP6, and the BMP2/6 what suppresses hepcidin by inhibiting hepatic BMP/SMAD signaling. Therefore, iron availability by stimulated erythropoiesis can be regulated by ERFE (9). Bone formation by osteoblasts during skeletal development, modeling, and ongoing remodeling can be stimulated by BMPs. Therefore, ERFE seems to be key in the recently described erythropoiesis-iron-bone circuit, by modifying the availability of BMP. Therefore, ERFE appears to be an important link between abnormal erythropoiesis, iron metabolism alteration, and loss of BMD in β-thalassemia (16, 17).

The mechanism of action of ERFE, through the sequestration of BMP, could be that the loss of ERFE, by improving the availability of BMP, stimulates the formation of osteoblastic bone (18). However, high ERFE levels are osteoprotective and prevent bone loss in β-thalassemia when erythropoiesis is extended. Therefore, there is a paradoxical effect that has not yet been fully explained. Although in TDT patients, in whom ERFE is inhibited post-transfusion, and is lower than in NTDT patients, could explain the more severe bone alterations in these patients despite transfusions and ERFE has a protective function decreasing bone loss phenotype in b-thalassemia.

In addition, other data indicate that the loss of BMP signaling (high ERFE), increases bone mass through direct inhibition of osteoclasts and activation of the Wnt pathway, predicting that the loss of ERFE would lead to a decrease in bone mass (16, 17) by increased expression of RANKL and sclerostin.

In summary, to date the only known function of ERFE was hepcidin regulation expression through BMP sequestration, contributing to iron overload (9). However, ERFE seems to have bone metabolisms implications and a new role in bone protection has been described (16). In conditions of elevated ERFE, such as β-thalassemia and others ineffective erythropoiesis situations, BMP2 and BMP6 proteins are sequestered, decreasing signaling through the BMP/Smad and ERK pathways. This would result in decreased SOST and RANKL expression (Rankl/OPG) with decrease osteoclastogenesis and bone resorption. On the other hand, when level ERFE is low, increased BMP2, and BMP6 proteins, lead to stimulate osteoclastogenesis (RANKL/Opg), bone resorption and increased sclerostin osteocytes synthesis (expression SOST gene), with a consequent decrease in bone formation by inhibition of osteoblastic function.

## GATA1 Levels Regulation in β-Thalassemia

As mentioned above, erythropoiesis starts from hematopoietic stem cells (HPSCs), in a finely regulated process, that involves various factors, and it is controlled at different molecular levels by growth factors and hormones such as erythropoietin, that activate different signaling pathways that end up activating erythroid transcription factors. The essential transcription factors for erythropoiesis are GATA-1, SCL, TAL1, LMO2, LDB1, KLF-1, and GFI-1B, although many more participate. These factors are organized in a complex called CEN (“Core ErythroidNetwork”) (19), whose operation is finely regulated by SCF and EPO between others, to ensure adequate erythrocyte development. GATA1 is considered the “master regulator” of this process, GATA1 is a DNA-binding zinc finger transcription factor that plays an essential role in the normal development of hematopoietic lines. The protein contains 413 amino acids, with an N-terminal region where its transcriptional activity resides and a C-terminal region that mediates the binding of GATA1 to DNA and other proteins. In 1995, two isoforms of GATA1 resulting from alternative splicing were identified. GATA1 encodes a 47 kDa protein and GATA1s, a shorter 40 kDa protein that lacks the transactivation domain at the N-terminus. Both proteins are capable of binding to DNA and could form dimers or heterodimers, although the shorter GATA1s isoform is less active than the long (20). In 1999 cleavage of GATA1 by Caspase 3 was reported (21). More recently, it has been linked the stabilization of GATA1 to inhibition of Caspase-1 Activity (22).

β-thalassemia shares several common elements with other forms of anemia including a deficiency of GATA1 the ‘master regulator' in erythropoiesis (23), GATA1 levels are finely regulated during erythropoiesis to develop sufficient functional erythrocytes (24). This transcription factor is necessary for normal early erythroid progenitors' differentiation [i.e., colony-forming unit-erythroid (CFU-E) and burst-forming unit erythroid (BFU-E) cells]. In β-thalassemia, α-hemoglobin chains accumulate in the cytosol due to the non-functionality of β-hemoglobin chains and sequester heat shock protein 70 (HSP70). Subsequently, HSP70 cannot be translocated into the nucleus thereby impairing GATA1 stabilization through caspase 3 cleavage, resulting in altered GATA1 levels with disturbed erythropoiesis and accumulation of unfunctional erythroid progenitors (23).

Importantly, GATA1 is highly expressed during these early stages of differentiation but GATA1 protein expression is shown to decline toward terminal erythroid differentiation (25). Recent data from Tyrkalska et al. demonstrated the role of the inflammasome, a complex of innate immune system which receptors and sensors playing important roles in infection and inflammation, in GATA1 regulation, and subsequent erythroid differentiation. Pharmacological inhibition of the inflammasome was shown to stimulate GATA1 expression and promote erythroid differentiation (22).

On the other hand, Fetal hemoglobin (HbF) increase has revealed as a promising results to treat β-hemoglobinopathies (26) with a recent implication of MiR-486-3p and miR-15a in Fetal hemoglobin induction (27). Several groups reported that increase of miR-210 levels is high in erythroid precursors from β-thalassemia patients what have an impact on fetal hemoglobin (HbF) levels (28–31).

Finally, other genetic studies indicate a relationship between variants in the gene BCL11A and HbF levels (29). There they described that reduced BCL11A expression induce HbF. The data published by Gasparello et al. are consistent with a globin gene regulation by BCL11A and therefore postulated BCL11A as a therapeutic candidate to reactivate HbF in disorders associated to beta-hemoglobin (29). Aligned with this, Bauer et al. published that GWAS-marked BCL11A enhancer represents another potential optional treatment in this disease (32).

**Figure 3:**
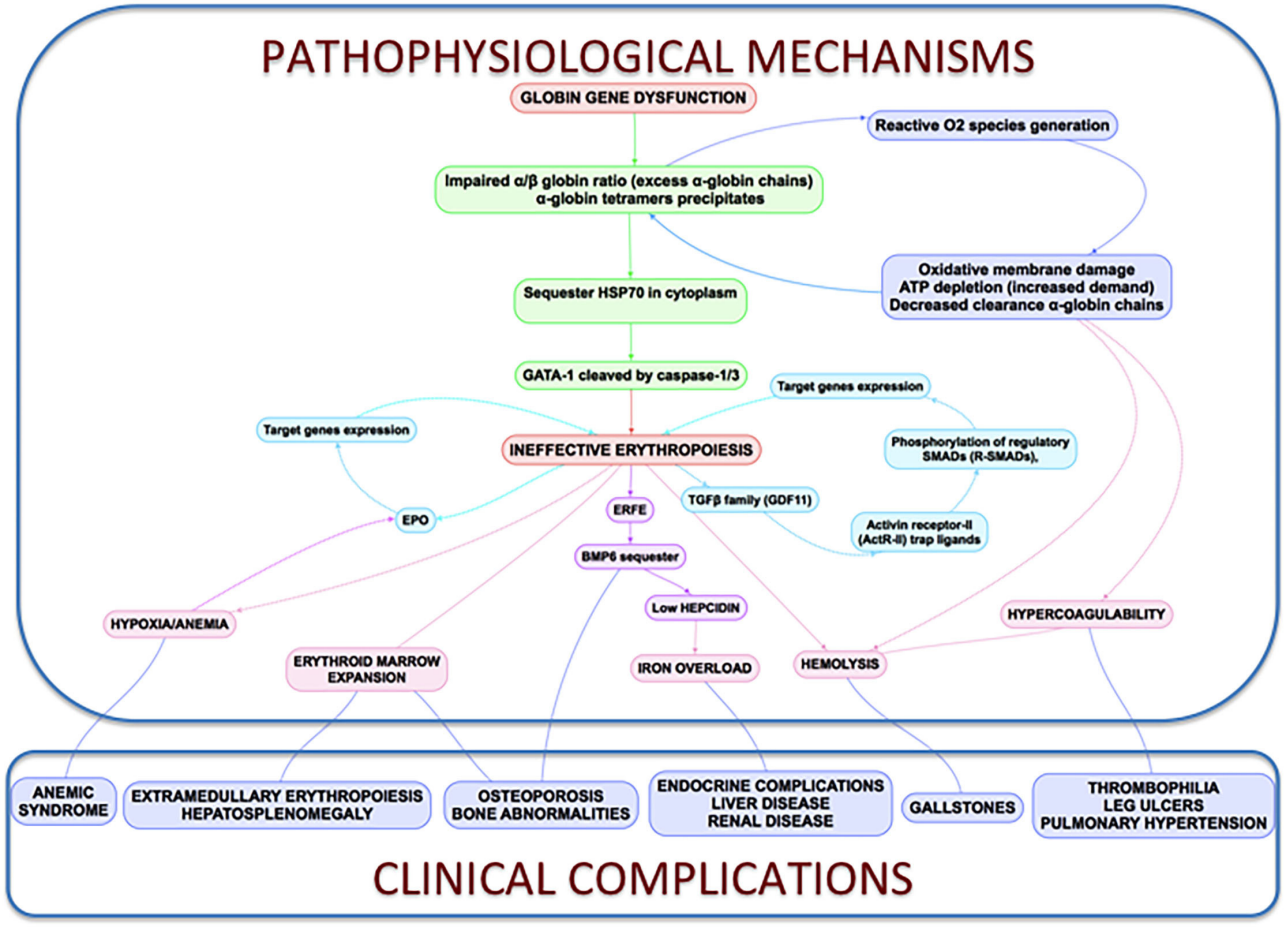
Clinical complications and pathophysiological mechanisms of β-Thalassemia. In thalassemia, the imbalance α/β-globin synthesis is the fundamental initial pathogenic event. Excess α-globin chains precipitate in the cytoplasm, sequester HSP70 and GATA1 is cleaved by Caspase 3/1 which result in dysfunctional erythropoiesis and imposes metabolic stress on the erythrocytes, specifically in the form of excess generation of reactive oxygen species and increased demand on adenosine triphosphate (ATP)-dependent proteolytic mechanisms to clear excess globin chains. These pathophysiological changes lead to the characteristics of this disease: ineffective erythropoiesis, peripheral hemolysis, and subsequent anemia. Clinical implications of the α- and β-globin imbalance include lack of sufficient RBCs and Hb for effective oxygen transport, and ineffective erythropoiesis and hemolysis, which can lead to splenomegaly, bone marrow expansion (extramedullary hematopoiesis), concomitant bone deformities, and iron overload.

All the processes described above are reflected and connected in Figure 3.

## Discussion: Perspectives and Future New Potential β-Thalassemia Treatments

Despite the high social and economic impact of β-thalassemia, there is no curative treatment available, except for bone marrow transplant for the few pediatric patients who have an identical HLA donor, and who assume the high morbidity and mortality of transplant that is sometimes not acceptable for non-malignant disease. Currently no approved treatments to handle anemia in NTDT and, even though it has recently been approved, the first drug that improves anemia in these patients (luspatercept), has limited efficacy. Therefore, there are no effective treatments to improve anemia or to reduce red blood cell transfusions and chronic complications. However, in recent years there is a growing interest in studying these diseases with an increasing number of clinical trials directed against various therapeutic targets (gene therapy, erythroid maturation agents, pyruvate kinase activators, JAK kinase 2 inhibitors, targeting iron dysregulation). All of them are being developed and in the future may change the quality of life of these patients and we aim to be part of this scenario. All of them summarized in a succinct way by Musallam et al. (33).

Although these targets may be effective, efficacy is partial, not all patients respond, and therefore further research and treatments are required considering its multifactorial pathophysiology. Perhaps the correct approach in the future is combined treatment and, in this scenario, it will be essential to explore new therapeutic targets. In recent years, inflammasomes have emerged as a new potential therapeutic target for these types of diseases as well as the HSP70 nuclei regulation.

### Inflammasome as a Potential Target Treatment in β-Thalassemia

Inflammasomes are multiprotein complexes firstly described a decade ago by Tschopp and colleagues. Inflammasomes are multiprotein complexes usually composed of sensor proteins, mainly from the NLR family, adaptor proteins such as ASC, and an effector cysteine-protease enzyme, usually caspase-1 (34). Gasdermin D (GSDMD) a pore-forming protein is cleavage and activated by caspase-1 (35), what induce cytokine release and pyroptosis (36). Several inflammasome receptors have been described with different roles including inflammatory diseases, sepsis protection or host defense (37–39).

Inflammasomes are broadly expressed in hematopoietic and non-hematopoietic cells and can induce different responses including production of IL-18, IL-1β, eicosanoids, and pyroptosis. Since this first description, research within the inflammasome field has been one of the most studied fields in immunology leading to huge advances.

In the innate immune context, the activation of inflammasomes is essential in the clearance of pathogens or damaged cells. On the other hand, uncontrolled inflammasome activation induces metabolic and autoimmune disorders, indicating the importance of these complexes. The recent role of the inflammasome in erythropoiesis brings the focus to these complexes to treat anemia and neutrophilia (22).

**Figure 4:**
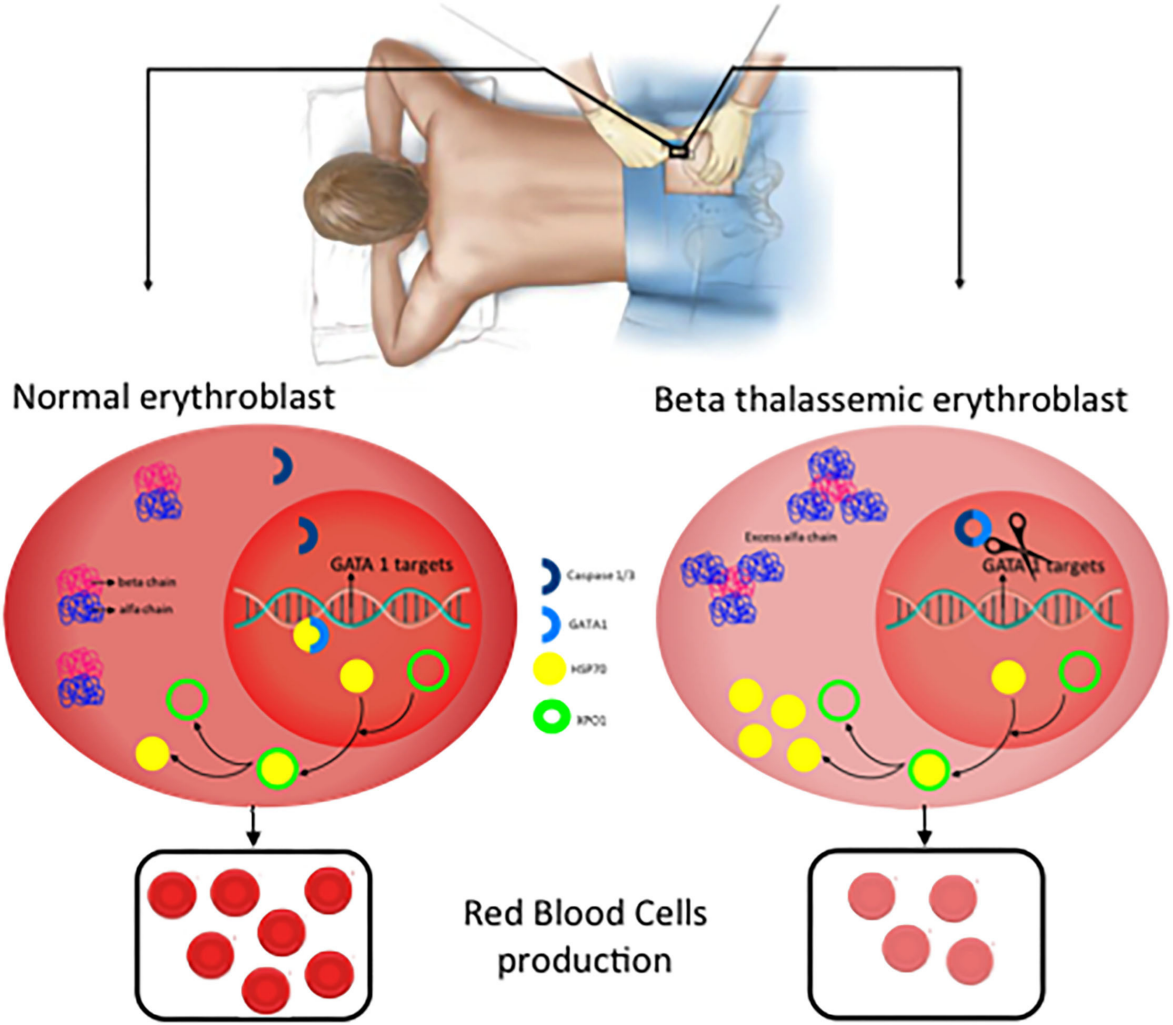
New potential treatments for β-thalassemia. In normal erythroblast, GATA1 levels are regulated through the balance Caspase 3/1 cleavage and HSP70 protecting function in the nucleus. As a consequence, normal and functional erythrocytes are produced. In contrast, in β-thalassemic erythroblast, the lack of functional β-globin chains induces accumulation of free α-globin chains which restrict HSP70 distribution to the cytoplasm and therefore GATA1 is cleaved by Caspase 3/1 which result in fewer functional matured erythrocytes.

The effector protein of the inflammasomes are caspases and previous data indicated a role of Caspase 3 in GATA1 stabilization (21). More recently, inflammasome has been postulated as a potential treatment of Diamond-Blackfan anemia, this type of anemia curses by a deficiency in GATA1 levels due to a ribosomopathy that produces an inefficient translation of GATA1 (22, 40). Tyrskalska et al. (22)demonstrated an increase of GATA1 levels in human and zebrafish larvae with caspase-1 inhibitor. Therefore, potentially inhibition of Caspase-1 or Caspase-3 could be considered as a potential treatment to stabilize GATA1 levels to increase red blood cells formation. In this scenario, the identification of the inflammasome type that mediates this process will be essential to translate these results with higher specificity to the clinic (Figure 4).

### Inhibition of HSP70 Export From the Nucleus

As described above, GATA1 levels are essential in erythroid differentiation, and in this plays an important role the Heat Shock Protein 70 (HSP70) a chaperone, which is translocated to the nucleus to protect GATA1 transcription factor of caspase-3 cleavage (41). In β-thalassemia, the accumulation of free α-globin chains sequestered HSP70 in the cytosol which avoids the protective role of GATA1 into the nucleus. A recent publication demonstrated that HSP70 localization is regulated by the exportin-1 (XPO1) and inhibition of XPO1 increase HSP70 levels in normal erythroid progenitors what increase, which have as a consequence an increase in GATA1 levels (42). This introducesXPO1 inhibitors as a new therapeutic option to treat β-thalassemia (42, 43).

For many years, management of β-Thalassemia patients has been limited to blood transfusion and iron chelation. However, β-Thalassemia is now the focus of a flourishing research field that has already offered new treatments with the potential to modify the natural history of the disease and the quality of life of the patients. In this review we have summarized the present knowledge of the pathophysiology of the disease and proposed future possible new, and more directed approaches, to its treatment (Figure 4).

## Conclusions

β-thalassemia (transfusion and non-transfusion dependent), is an inherited hemoglobinopathy caused by a quantitative defect in the synthesis of β-globin chains of hemoglobin, leading to the accumulation of free α-globin chains aggregates that cause ineffective erythropoiesis. The only curative treatment for these patients is hematopoietic stem cell transplantation, but this option only is feasible in a few patients with HLA-matched sibling donors. In most patients, the development of chelation and support treatments has improved survival, however, chronic complications have increased (iron overload, osteoporosis, extramedullary hematopoiesis, etc.) that limit their quality of life.

Despite increasing knowledge of the pathophysiology of β-thalassemia, only luspatercept has been recently approved in TDT patients, reducing transfusion needs but of limited effectiveness. NTDT patients, which do not require regular transfusions, lack an approved treatment and have the same (if not more) complications as TDT patients. Therefore, new treatments are needed that, alone or in combination with existing ones, can improve the expectations of these patients.

In human erythroblast, terminal erythroid maturation is altered due to HSP70 sequestration in the cytoplasm by free α-globin chains preventing its accumulation in the nucleus to protect GATA1 transcription factor from Caspase-3 cleavage, resulting in maturation arrest and apoptosis. ERFE and BMP play an important role in bone disease but are not well-established yet.

The knowledge of these critical new pathophysiological approaches can help develop new therapeutic options such as XPO1 inhibitors or inflammasome inhibitors that could rescue GATA-1 expression, improved erythroid terminal differentiation, and represent a new therapeutic option to ameliorate ineffective erythropoiesis, iron overload, and decreased mineral bone mass of β-thalassemia patients.

## Data Availability Statement

The datasets presented in this study can be found in online repositories. The names of the repository/repositories and accession number(s) can be found in the article/supplementary material.

## Author Contributions

MS-V: writing—original draft preparation. AP-O, ES, JM, and MB: writing—review and editing. All authors have read and agreed to the published version of the manuscript.

## Funding

This work was supported by the Institute of Health Carlos III (ISCIII) through a Miguel Servet Contract Program and the Associated Budget CP20/00028 to AP-O partially funded by European Funding. The funders had no role in the study design, data collection and analysis, decision to publish, or preparation of the manuscript.

## Conflict of Interest

The authors declare that the research was conducted in the absence of any commercial or financial relationships that could be construed as a potential conflict of interest.

## Publisher's Note

All claims expressed in this article are solely those of the authors and do not necessarily represent those of their affiliated organizations, or those of the publisher, the editors and the reviewers. Any product that may be evaluated in this article, or claim that may be made by its manufacturer, is not guaranteed or endorsed by the publisher.
